# Centriolar satellites prevent uncontrolled degradation of centrosome proteins: a speculative review

**DOI:** 10.15698/cst2018.02.122

**Published:** 2018-01-24

**Authors:** Nicolas Lecland, Andreas Merdes

**Affiliations:** 1 Centre de Biologie du Développement, Université Paul Sabatier/CNRS, 31062 Toulouse, France.

**Keywords:** centriolar satellites, centrosome, ciliogenesis, protein degradation, chaperone

## Abstract

Centriolar satellites are small electron-dense structures in the cytoplasm, mostly surrounding the pericentriolar material. Initially viewed as shuttles for the transport of centrosomal proteins, they have been implicated in the assembly of the pericentriolar material and in ciliogenesis. Although numerous proteins have been identified as components of centriolar satellites, their molecular function remains unclear. In this review article, we discuss recent findings that characterize centriolar satellites as regulators of protein degradation pathways: by sequestering E3 ligase MIB1, deacetylase HDAC6, and proteins of the autophagy pathway, centriolar satellites may regulate the turnover of centrosomal and ciliary components, protecting them from removal via proteasomal degradation, autophagy, and aggresomes.

## INTRODUCTION

Centrosomes are cytoplasmic structures that serve as primary microtubule organizing centers in a large variety of animal cells. They consist of a pair of cylindrically shaped centrioles, surrounded by pericentriolar material. Since the 1950s, an impressive amount of light and electron micrographs have been published that document the structure and molecular composition of centrosomes. Early on, scientists noted the existence of small electron-dense granules in the cytoplasm surrounding the centrosome, within a radius of several microns from the centriolar cylinders. Because of their size, structure, and localization, these granules were occasionally named "satellites", or "pericentriolar dense bodies", since the term "satellites" was also used to describe subdistal appendages of centrioles 1,2. The functional characterization of these granules was fueled by the discovery of their first molecular marker, PCM-1 (originally named after "pericentriolar material 1"). Immunofluorescence showed that PCM-1 localizes to the pericentriolar material in interphase, and to small puncta in the surrounding cytoplasm 3. In mitosis, the pericentriolar accumulation of PCM-1 is temporarily lost. Immunoelectron microscopy revealed that PCM-1-containing centriolar satellites are non-membranous structures of 70 to 100 nm diameter that accumulate not only at the periphery of centrioles, but also near the basal bodies in ciliated cells 4. Satellite-like structures that don’t contain PCM-1 have also been reported for various centrosome proteins, visible by immunofluorescence as small cytoplasmic foci in the neighborhood of the centrosome 5. Because the molecular composition of centriolar satellites has been reviewed extensively in previous publications 6,7,8, this article focuses on satellites containing the marker protein PCM-1, and on the potential molecular functions of PCM-1 and its binding partners within these structures.

## CENTRIOLAR SATELLITES AS SHUTTLES FOR THE TRANSPORT OF CENTROSOMAL PROTEINS

Early studies showed that PCM-1 satellites move along microtubules, towards and away from centrioles 4. It was shown that the dynein/dynactin motor complex is involved in the centripetal movement of the satellites, and that part of the PCM-1 satellites co-localize with cytoplasmic foci of various centrosome proteins 4,5,9. Since siRNA-mediated depletion of PCM-1 led to reduced accumulation of centrin, pericentrin, and ninein at the pericentriolar material, PCM-1-containing satellites were viewed as shuttles that transport centrosome components from the cytoplasm to the surface of the centrosome 5. Other proteins whose centrosomal accumulation is facilitated by PCM-1 include gamma-tubulin, Nek2, and C-Nap1 9,10,11. However, the simple concept of PCM-1 satellites as shuttles for centrosomal protein transport falls short to explain why only part of the cytoplasmic foci of centrosomal proteins co-localize with PCM-1 satellites, and why loss of shuttle activity isn’t compensated by simple diffusion, since proteins such as centrin are 90% soluble in the cytoplasm 12.

## CENTRIOL SATELLITES AND CILIOGENESIS

Because PCM-1-containing satellites were seen highly enriched near basal bodies in ciliated cells, a role of PCM-1 in ciliogenesis had been suspected 4. Further research indicated that PCM-1 satellites interact with proteins of the BBSome, a complex of seven conserved proteins that are encoded by susceptibility genes of the "Bardet-Biedl Syndrome" (BBS) and that are involved in membrane trafficking to the primary cilium 13,14. Silencing of PCM-1 dislocated two interacting proteins, Cep72 and Cep290, from the satellites to the pericentriolar material, and various silencing experiments indicated that PCM-1, Cep72, and Cep290 are needed for the targeting of the small GTPase Rab8 and of BBS proteins to the primary cilium 15,16. Finally, these experiments showed the need of intact centriolar satellites for efficient ciliogenesis. Although an involvement of centriolar satellites in the transport and delivery of ciliary components could not be excluded, the molecular function of PCM-1 in this process remained unclear. To shed light on this, we are reviewing a variety of observations that indicate less known mechanisms by which satellites may regulate ciliogenesis and centrosomal function.

Several years ago, a novel perspective on the function of PCM-1 emerged from a phylogenetic analysis of centrosomal proteins in 45 organisms, representing a wide evolutionary spread across the major groups of eukaryotes. A phylogenetic profile demonstrated a correlation between the evolution of ciliary photoreceptor cells in the eye and the presence of BBSome components and of PCM-1 17. Photoreceptor cells in the eye of vertebrates contain light-sensitive outer segments that represent modified cilia, with stacked membrane disks involved in phototransduction. These photoreceptor cells are not renewed during lifetime, but internal protein turnover assures their viability. This turnover involves autophagy and massive transport of outer‐segment material through the connecting cilium 18. Since autophagy protects photoreceptor cells from damage after exposure to strong light stress, and since autophagic activity follows periods of reduced illumination in the circadian rhythm or in hibernation in various animals 19,20, it may be speculated whether proteins such as PCM‐1 are involved in light-dependent degradation and renewal of ciliary outer segments. Since PCM-1 is present in nearly all cell types in vertebrates, the more general question arises as to whether PCM-1 and centriolar satellites are linked to degradation and protein turnover during the formation and maintenance of cilia.

## CENTRIOLAR SATELLITES AND AUTOPHAGY

**Figure 1:**
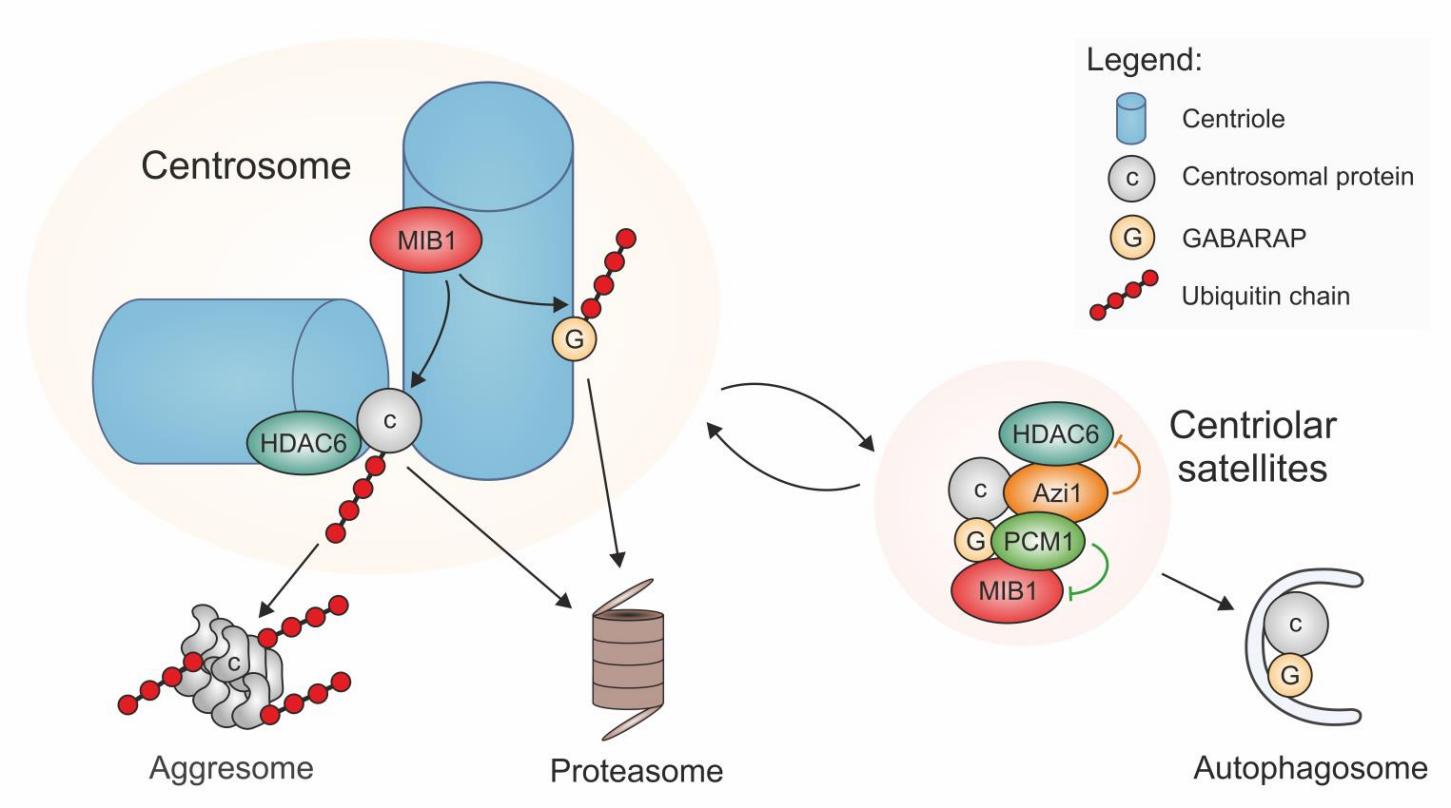
FIGURE 1: Two core components of centriolar satellites, PCM-1 and Azi1/Cep131, sequester E3 ligase MIB1, autophagy component GABARAP, and deacetylase HDAC6, to inhibit removal and degradation of centrosome proteins by proteasomes and aggresomes, and to enable controlled degradation by autophagy. Cargoes of the centriolar satellites are in a dynamic exchange with the pericentriolar material. If bound to the pericentriolar material, MIB1 mediates poly-ubiquitylation (red) of proteins that are destined to proteasomal degradation or to HDAC6-mediated accumulation in aggresomes. Among these MIB1-substrates may be proteins of the pericentriolar material (c, grey), or centrosome-bound regulators of the autophagy pathway, such as GABARAP (G, dark yellow). Degradation of specific centrosome proteins, such as Ofd1, may occur via the autosomal pathway, supported by satellite-mediated pickup of these proteins together with GABARAP, and delivery to early autophagosomes.

Indeed, data published in recent years suggest that PCM-1-containing centriolar satellites may be at the crossroad between proteasomal and autophagic degradation pathways. Proteomic analyses and localization studies revealed that PCM-1 forms a multiprotein complex with Azi1/Cep131 and the E3 ubiquitin ligases MIB1 and WWP2, and associates with numerous other centrosomal proteins, often in a transient manner 6,7,8,21,22,23. Moreover, PCM-1 interacts with multiple proteins involved in autophagy, such as LC3, GATE16, and GABARAP 24. The latter are proteins of the Atg8 family that recruit cargoes to autophagosomes for subsequent lysosomal degradation. One cargo that has been characterized in the context of ciliary function is Ofd1, a negative regulator of ciliogenesis that binds to the centrosome and to basal bodies 25. To enable the formation of a new primary cilium, Ofd1 needs to be eliminated. This is accomplished by an interaction of Ofd1 with PCM-1 at centriolar satellites, from where Ofd1 is degraded by the autophagosome-lysosome pathway 24,26. In this process, centriolar satellites may regulate Ofd1 removal by controlling the levels of Atg8 proteins, such as GABARAP. This occurs in two ways: 1) GABARAP accumulates at the pericentriolar material, where its abundance is limited by MIB1-dependent polyubiquitylation, followed by proteasomal degradation 27. PCM-1-dependent sequestration of MIB1 to satellites suppresses this polyubiquitylation 27, and may therefore help with fine-tuning the protein levels of GABARAP. 2) At the same time, PCM-1 can bind to GABARAP and other proteins of the Atg8 family (LC3, GATE16, and others) by an LIR (LC3-interacting region) sequence motif near the PCM-1 carboxy-terminus. With the help of the LIR motif, PCM-1 mediates both the association of GABARAP with centriolar satellites, and its delivery to the pericentriolar material. The pool of pericentriolar GABARAP is in an equilibrium with GABARAP bound to satellites, and satellite-bound GABARAP is believed to be in an inactive state 27. The presence of the LIR motif in PCM-1 is directly responsible for the maintenance of regular GABARAP protein levels 27, suggesting that binding to PCM-1 protects GABARAP. Upon serum starvation, satellites that contain PCM-1 and Atg8 proteins associate with early autophagosomes and transfer the ciliogenesis inhibitor Ofd1 together with the Atg8 protein into these organelles (**Figure 1**). The autophagic removal of Ofd1 activates ciliogenesis. PCM-1 is thus involved in the flux of components and cargoes towards early autophagosomes, but it has no role further downstream in the autophagic process 24,27.

## CENTRIOLAR SATELLITES AND THE PROTEASOME

A link between PCM-1 and proteasomal degradation has first been established by Didier *et al*. 28. Treatment of cells with proteasome inhibitors leads to massive accumulation of various centrosome proteins at the pericentriolar material, and to an enrichment of PCM-1-containing centriolar satellites in the proximity of the centrosome. This suggests that there is constant turnover of proteins of the pericentriolar material, involving proteolytic degradation. The discovery of the E3 ubiquitin ligases MIB1 and WWP2 as interactors of PCM-1 21,22 raises the question whether centriolar satellite components mediate directly the ubiquitylation of centrosomal proteins prior to delivery to proteasomes, since inhibition of proteasomal activity creates a backlog of centriolar satellites and of polyubiquitylated, but undestroyed centrosomal proteins 21,22,28. However, closer investigation revealed that the major sites of MIB1-dependent polyubiquitylation are at the centrioles, not at the centriolar satellites, and that PCM-1 prevents polyubiquitylation and proteolysis by sequestering MIB1 to the satellites, as already discussed in the context of GABARAP and autophagy regulation (**Figure 1**) 27,29. As a prominent example for this MIB1-sequestration mechanism, PCM-1 was found to prevent the polyubiquitylation and destruction of the centriolar protein talpid3 29. Since talpid3 is needed to associate with Rab8, to enable the recruitment of ciliary vesicles, the PCM-1-dependent restraint of MIB1 activity helps to promote ciliogenesis 29.

Under normal circumstances, polyubiquitylation of centrosomal proteins by centriole-bound MIB1 may serve to eliminate misfolded protein, or it may be part of a regulatory network to ensure homeostasis of the pericentriolar material, i.e. to maintain constant protein amounts by removing surplus protein. In this context, satellites may be fine-tuning this activity by preventing excessive removal.

## CENTRIOLAR SATELLITES AND HDAC6

Centriolar satellites may regulate protein degradation at yet another level: the satellite component Azi1/Cep131 possesses a putative HDAC-interacting domain that should permit binding to deacetylases, such as HDAC6 30. In addition to its deacetylase activity, HDAC6 has the ability to bind poly-ubiquitylated proteins and to transport these to aggresomes, by interacting with the dynein motor complex 31. Aggresomes are structures near the microtubule-organizing center that accumulate poly-ubiquitylated misfolded proteins when the degradation capacity of proteasomes is exhausted. The presence of HDAC6 is required for the resorption of cilia. This resorption may be a measure to regulate ciliary signaling, or to permit entry into the cell cycle, or to respond to cellular stress following heat shock 32,33,34. For this activity, HDAC6 localizes to the pericentriolar matrix of the centrosome/basal body, where it is activated by the kinase Plk1, whose centrosomal accumulation depends on the centriolar satellite component PCM-1 34. The HDAC6-mediated transfer of proteins to aggresomes may be limited by the satellites, in a manner similar to the quenching of E3 ligase activity of MIB1: HDAC6 may be removed from the active centrosomal pool by sequestering it to satellites. In this way, centriolar satellites may contribute to ciliogenesis, by inhibiting ciliary resorption.

## CONCLUSION

Until today, a large number of proteins have been identified as potential interactors of centriolar satellite components. Many of these proteins need to accumulate at the pericentriolar material of centrosomes or basal bodies, to fulfil their function in microtubule organization or in ciliogenesis. Centriolar satellites can support their accumulation indirectly: although the satellites may be able to transport specific cargo to the pericentriolar material, their more important role may be to protect centrosomal proteins from degradation, by sequestering proteins of several degradation pathways into satellites, such as E3-ubiquitin ligases, autophagy components, and deacetylases. Satellite components PCM-1 and Azi1/Cep131 may keep these degradation factors temporarily bound, in an inactive state. Controlled release of these factors may contribute to maintain constant levels of centrosomal proteins, by permitting removal of excess protein from the pericentriolar material, or by controlling the replacement of misfolded protein by intact protein. Loss of PCM-1 leads to release of degradation factors, provoking polyubiquitylation and degradation of centrosome proteins. PCM-1 and Azi1/Cep131 exist in two pools that are in exchange, at the pericentriolar material and in centriolar satellites 4,35. This may allow autoregulation of satellite activity, by MIB1-dependent ubiquitylation of PCM-1 and Azi1/Cep131 at the centrosome 22,29, counterbalanced by USP9X, a deubiquitinase that stabilizes both proteins 36,37.

Future studies will be necessary to find out how proteins are sequestered into centriolar satellites, and how satellite function is regulated. Structural analysis may help to determine whether PCM-1 and Azi1/Cep131 have similarities to molecular chaperones, since they can keep binding partners such as MIB1 or GABARAP temporarily bound to satellites, before releasing them in an active state. Moreover, mutants of PCM-1 and Azi1/Cep131 should be generated to test whether their activity requires assembly into satellites, or whether they remain functional outside these structures. Data from specialized cell types in animal tissues demonstrate that the localization of PCM-1 can alter during differentiation, for example from pericentriolar satellites to the cytoplasmic surface of the nuclear envelope in differentiating myoblasts 38, suggesting that satellite markers may serve additional roles outside centriolar satellites that may be unrelated to centrosomal or ciliary function.

## Abbreviations

BBS: Bardet-Biedl Syndrome
LIR: LC3-interacting region
